# Maxilla–nasion–mandible (MNM) angle: an indicator to assess fetal facial profile in first-trimester of pregnancy

**DOI:** 10.1186/s40064-016-2944-4

**Published:** 2016-08-11

**Authors:** Wei Liu, Suhui Qu, Mujun Wang, Wanju Xu, Guangying Zhang, Chengqi Zhang

**Affiliations:** 1 Department of Ultrasound, Shandong Provincial Qianfoshan Hospital, Shandong University, Jinan, 250014 Shandong China; 2 Department of Surgery, The First People’s Hospital of Jinan City, Jinan, 250011 Shandong China; 3 Department of Clinical Laboratory, Shandong Provincial Qianfoshan Hospital, Shandong University, Jinan, 250014 Shandong China; 4 Department of Medicine Imaging, Shandong Provincial Qianfoshan Hospital, Shandong University, No. 16766 Jingshi Road, Jinan, 250014 Shandong China

**Keywords:** MNM, Aneuploid, Micromandible, Trisomy 18, Chromosome

## Abstract

**Objective:**

The aim of this study was to observe whether there existed significant differences in the maxilla–nasion–mandible angle (MNM) between the first- and second-trimester of pregnancy, and to observe its predictive values for trisomy 18.

**Methods:**

Two experienced ultrasonologists used 2D and 3D ultrasound imaging techniques to obtain the facial sagittal sections of fetuses in the first-trimester of pregnancy (crown-rump length 45–84 mm), respectively, so as to measure MNM.

**Results:**

MNM could be measured in 91 % of normal fetuses, and the measurement differences by different operators in different groups were <1.1°; average MNM was 11.0°, and no significant change was observed in different gestational ages (*P* = 0.15). The average of MNMs in fetuses with trisomy 18 was 16.6°, which were all higher than the 95th percentile of normal measurement data. The sensitivity and specificity of increased MNM on the abnormal detection of trisomy 18 were 54.7 and 97.4 %, respectively.

**Conclusions:**

The feasibility and reproducibility of measuring MNM in early pregnancy were good. MNM had certain suggestive roles for aneusomic chromosomal abnormalities, especially for fetuses with trisomy 18.

## Background

The clinical manifestations of autosomal aneuploidy abnormality (AAA) mainly include mental retardation and multi-organ abnormalities such as face and heart. The low survival rate of the fetuses with this disease brought great social and family burdens (Nyberg and Souter 2003; Parker et al. 2010; Cereda and Carey 2012), so it has always been the focus of prenatal diagnostic screening. Presently, the main screening method towards aneuploid is to detect the nuchal translucency (NT) in early pregnancy combined with laboratory tests, as well as to screen fetal systems and structures in the second-trimester of pregnancy (Ekelund et al. 2011; Baer et al. 2015; Dovev et al. 2014). Studies had shown that 90 % of aneuploid in the second-trimester of pregnancy had ultrasonographic changes (American College of Obstetricians and Gynecologists ; Baer et al. 2015; Dovev et al. 2014; Wagner et al. 2015); however, in early pregnancy, because fetuses are small, and their structural developments are imperfect, the detection is not ideal simply depending on ultrasonographic changes. Currently, the indicators with confirmed significance are only NT and nasal bone (Malone et al. 2005; Irving et al. 2011). How to improve the detection rate of fetuses with abnormal aneuploid in early pregnancy is worth further studies.

AAA has typical facial features, such as a flat face, micromandible, etc. Vos and de Jong-Pleij confirmed that maxilla–nasion–mandible angle (MNM) was one good indicator to assess fetal facial profile abnormalities and to diagnose special facial anomalies in middle and late pregnancy (Vos et al. 2012, 2015a), and the conclusion that MNM changed the with gestational ages was also drawn (Vos et al. 2015). Vos also reported that MNM had certain suggestive roles towards the screening of trisomy 21 and trisomy 18 in the second-trimester of pregnancy (Vos et al. 2015a, c, d; De Jong-Pleij et al. 2010). Therefore, it could be deduced that MNM could also be used as a good indicator to evaluate special facial abnormalities in early pregnancy. Targeting this deduction, this study observed the significance of MNM as an indicator to evaluate facial profile in early pregnancy, as well as whether it might have clinical significance towards the screening of AAA fetus.

## Methods

### Subjects

A total of 5300 fetuses in their early pregnancy (crown-rump length 45–84 mm) were screened in our hospital from January 2013 to December 2015. The 2D ultrasound and 3D volume collection were performed targeting the fetal head standard sagittal section, and the fetal head images were magnified to at least 1/3 of the screen to measure MNM. This study was conducted in accordance with the declaration of Helsinki. This study was conducted with approval from the Ethics Committee of Shandong University. Written informed consent was obtained from all participants.

### Measurement criteria

**Fig. 1:**
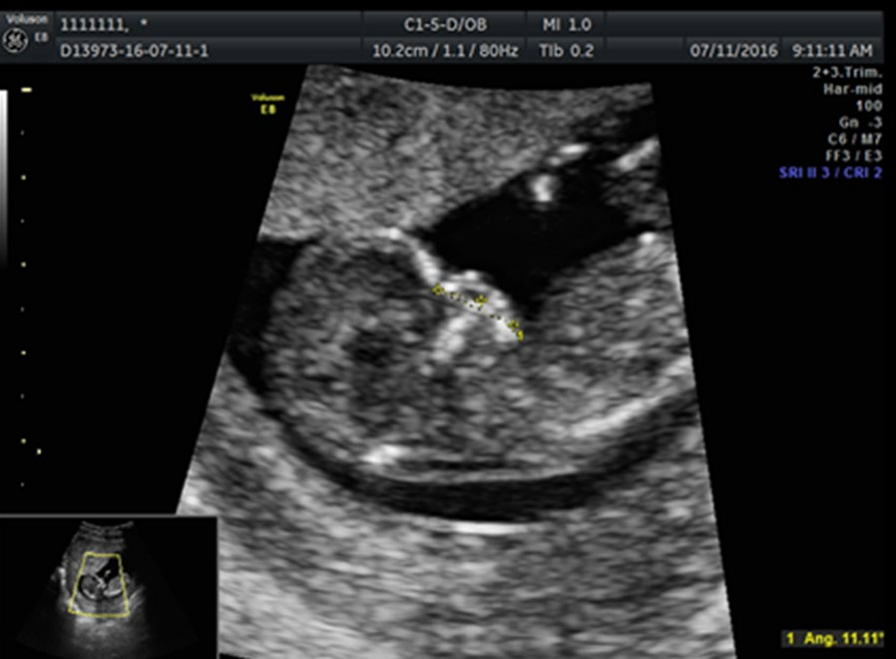
Measurement of MNM at the fetal median sagittal section in normal fetal

The multi-plane mode could much more accurately display the sagittal section, and only the real fetal facial median sagittal section images were selected for the analysis using the 4D view software. In this study, facial profile image should display the forehead, nasal bone, and mandible, and the maxilla should be displayed as a single horizontal line without jugal bone or mandibular branch. Definition of MNM: the angle between the maxilla–nasion line and the mandible–nasion line (Fig. 1). The nasion was defined as the intersection point of the foremost frontal bone and nasal bone; if there existed a ditch between the anterior frontal edge and nasal bone, the mark of the nasion was placed at the intersection point of the nasal tangent and the lowest forehead tangent. The marks of maxilla and mandible were defined as the midpoints of the foremost maxillary and mandibular edges. The ruler was placed at the outermost edge of the bony structure.

In order to ensure the repeatability, all the marks in all cases were completed by two experienced ultrasonologists under the conditions of not knowing the gestational age and the other ultrasonologist’s measurement data. The measurement data referred to the MNM range in the second-trimester pregnancy proposed by Vos, and any value lower than the 5th percentile or higher than 95th percentile of the relevant range was considered as abnormal (Vos et al. 2015a, c; de Jong-Pleij et al. 2011).

### Statistical analysis

The t test was used to analyze the difference among the measurement values, with *P* < 0.05 considered as statistically significant. EXCEL2003 and SPSS19.0 were used to analyze the data.

## Results

MNM was obtained from a total 4823 fetuses (91 %), and the rest fetuses could not be measured due to fetal over-flexion, nasal bone deletion, or poor maxillary display. There was no significant difference in the measurement values between the two ultrasonologists (*P* = 0.94).

**Fig. 2:**
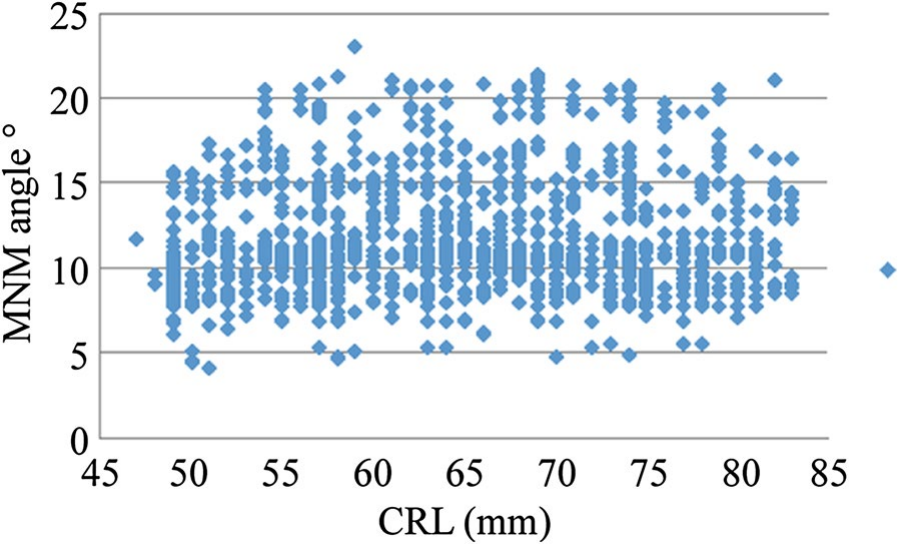
Scatterplot of MNM measurements in different gestational weeks

**Fig. 3:**
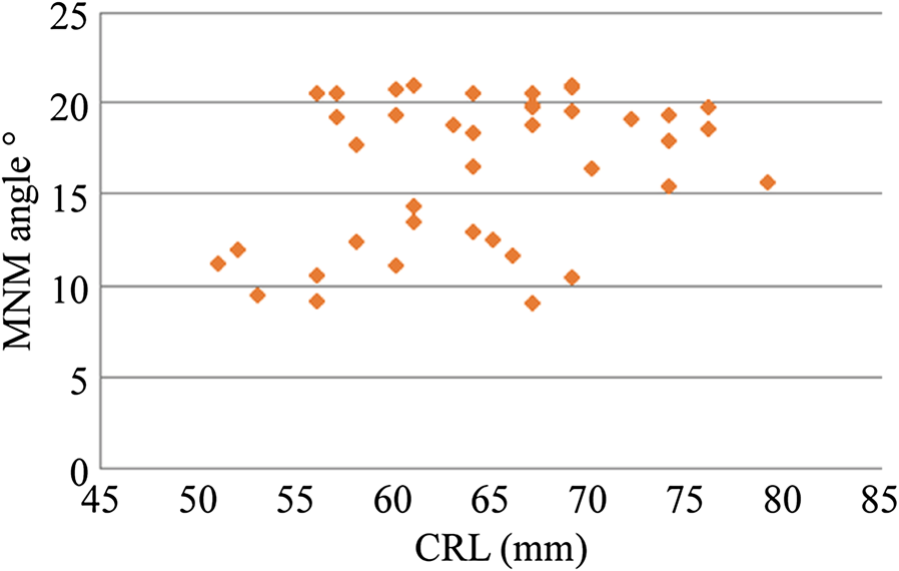
Scatterplot of MNM measurements in fetal with trisomy 18

The average MNM in the study subjects (4823 fetuses) was 11.00° ± 2.58° SD (95 % CI 10.29°–11.70°) (Fig. 2). The 5th and 95th percentiles were 6.79° and 15.02°. The gestational age and MNM showed no consistency (*P* = 0.15). There was no statistical significance between the measurement data of this study and Vos et al. (2011) (*P* = 0.62). Referring to the MNM range in the second-trimester pregnancy proposed by Vos et al. (2015a, b), a total of 42 cases of trisomy 18 were analyzed efficiently. Furthermore, MNMs in fetuses with trisomy 18 were all higher than the 95th percentile of the measurement data in this study (15.02°) (Fig. 3). The sensitivity of increased MNM on the abnormal detection of trisomy 18 was 54.7 %, and the specificity was 97.4 %.

## Discussion

Fetuses with AAA normally have typical facial features, such as flat face, micromandible, etc. Targeting the issue that whether these typical facial features could be used to improve the screening rate of AAA, certain researchers had proposed such facial indicators as the mandibular length, mandibular transverse diameter, mandibular anteroposterior diameter, etc. (Feuchtbaum et al. 2000), which improved the diagnosis of micromandible and retrognathia, but it’s rarely used clinically. One reason is that it requires the visualization of fetal facial axial and coronal planes, which are conventionally uneasy; in addition, it is very difficult to determine the locations of head marks, and the measurement of these indicators might be affected by acoustic shadows. In recent years, Vos and de Jong-Pleij confirmed that MNM was one good indicator to assess fetal facial profile abnormalities and to diagnose special facial anomalies in middle and late pregnancy (Vos et al. 2012, 2015a). The feasibility, repeatability, measurement difference in the second-trimester pregnancy, and significance in AAA screening of MNM in early pregnancy were confirmed in this study.

**Fig. 4:**
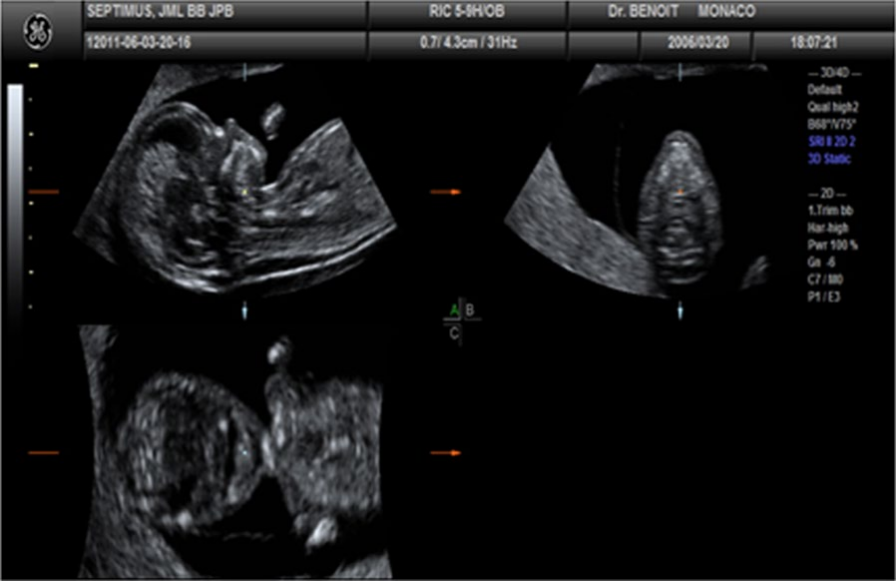
3D multi-plane imaging technique helped to obtain standard median sagittal section

The maxilla develops from the first branchial arch, including the former maxilla, palatine bone, zygomatic bone, and temporal bone, and all the above was developed through intramembranous ossification. In the 8th week of embryo development, the maxillary strip-like cell aggregation region outside the nasal sac begins the ossification; if the anterior maxilla forms a separate ossification center, it will soon fuse with the maxillary ossification center. Therefore, in early pregnancy (crown-rump length 45–84 mm), measuring MNM is theoretically feasible, which could also avoid the possibilities of missed-diagnosis and misdiagnosis due to poor ossification. In the practice of this study, the measurement marks of MNM in early pregnancy, namely the maxilla, nasion, and mandible, could be easily displayed in fetal sagittal section, and would only fail under the conditions of head over-flexion, poor maxilla–mandible display, or non-nasion. Furthermore, the 3D multi-plane ultrasound imaging could help us to better access the median sagittal section (Rotten et al. 2002) (Fig. 4), thus further ensuring the feasibility and accuracy of MNM measurement.

In addition, the frontal and nasal bones are not dependent, and the foremost edge of the lowest frontal bone would not be affected by the nasal bone, so when the nasal bone is short, the mark of the nasion and the lowest edge of the foremost frontal edge could use the intersection point of the nasal tangent and the lowest forehead tangent, and this would not affect the measurement of MNM. Therefore, no matter theoretically or practically, MNM could be used as a feasible method to evaluate fetal dysgnathia in the first-trimester pregnancy.

In this study, two experienced ultrasonologists performed the measurements independently, and there existed no significant inter- or intra-group difference, indicating the MNM measurement had a good repeatability.

This study showed no consistency between the gestational age and MNM, consistent with Vos. However, this study showed that the average MNM in early pregnancy was 11.00°, smaller than that in the study of Vos et al. (2012, 2015a) (13.5°), but there was no significant difference. The specific reasons still required further large-sample studies for the analysis.

Facial profile developments in fetuses and adults are different. In the fetal period, the maxilla and skull are anatomically fused, and are pushed forwards with the brain development, but the mandible is only connected with the skull by the temporomandibular joint. When the cranial level becomes protruding, in might imply difficulties for the mandible to catch up with the maxillary development, thus appearing retrognathia. Vos and de Jong-Pleij confirmed that MNM could be used to improve the diagnosis rate of contraction in late pregnancy, as well as the values of measuring facial concavoconvex angles (Vos et al. 2015d; de Jong-Pleij et al. 2011, 2013). The reduction of MNM might imply flat facial profile, which might be caused by maxillary hypoplasia or prorsad disappearing. These morbid states could be easily recognized in late pregnancy, but if there is no standard reference in early pregnancy, the judgment would still be difficult. This study confirmed that MNM could be used as the reference for determining the facial profile in early pregnancy.

**Fig. 5:**
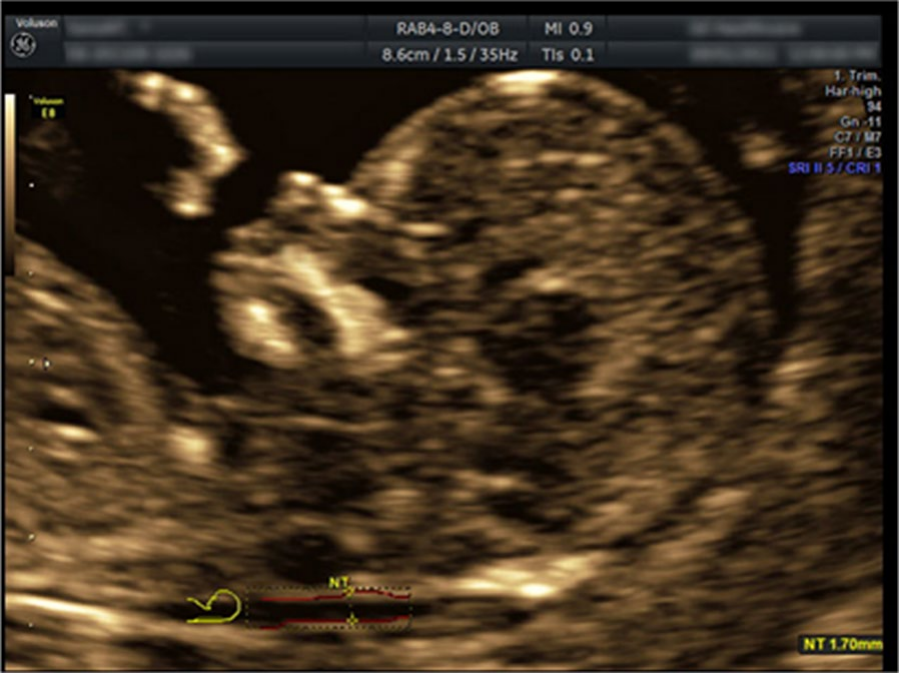
The imaging of the angle of MNM with significantly increased

This study showed that MNMs in the trisomy-18 fetuses with micromandible were all significantly increased, greater than the 95th percentile (Fig. 5). Because the anterior mandibular edge was used as a marker for the MNM measurement, it suggested that micromandible and non-micromandible could be technically verified by MNM. Similarly, factors causing the forward-shift of maxilla could also be confirmed by MNM. Therefore, MNM could be used as a method with forward direction for the early diagnosis of micromandible, opisthognathy, or alveolar fracture in early pregnancy. Similarly, flat forehead and maxillary hypoplasia caused abnormal forward shift would exhibit smaller MNM. In addition, as a reference point, when the forehead has such frontal deformities as forehead lump, it would affect the MNM measurement. So, it would still be necessary to investigate large samples to establish the exact MNM in early pregnancy towards diagnosing special facial anomalies.

In summary, MNM was a potential tool that could objectively evaluate the anteroposterior relations of maxilla and mandible in early pregnancy so as to assess the fetal facial profile. This tool had the potentials to help the prenatal understanding, classification, and diagnosis of fetal profile abnormalities (especially micromandible), thus helping the early screening of special facial features-related diseases.
